# Recurrent hyperkalemia in patients with chronic kidney disease and hepatitis C treated with direct antiviral agents

**DOI:** 10.1186/s12879-019-4117-x

**Published:** 2019-06-21

**Authors:** Taotao Yan, Jiuping Wang, Juan Li, Shan Fu, Yi Chen, Chunhua Hu, Rou Zhang, Zhen Tian, Fahui Zhao, Jun Dong, Jinfeng Liu, Yuan Yang, Tianyan Chen, Yingren Zhao, Yingli He

**Affiliations:** 1 Institution of Hepatology, First Affiliated Teaching Hospital, SOM, Xi’an Jiaotong University, Xi’an City, China; 2 Department of Infectious Diseases, First Affiliated Teaching Hospital, SOM, Xi’an Jiaotong University, No. 277 Yanta Road(w), Xi’an City, Shaanxi Province China; 3 Department of Infectious Diseases, Xijing Hospital of the Air Force Medical University, Xi’an City, China; 4 Department of Internal Medicine, Zhen’An County Hospital, Zhen’An, China; 5 Department of Haemodialysis, Zhen’An County Hospital, Zhen’An, China

**Keywords:** Chronic kidney disease, Sofosbuvir, Hyperkalemia

## Abstract

**Background:**

Sofosbuvir is the keystone of direct antiviral agents for the chronic hepatitis C (CHC). The safety of sofosbuvir in patients with stage 4–5 chronic kidney disease (CKD) needs further observation in real world.

**Case presentation:**

Thirty-three patients with stage 5 CKD and hepatitis C virus (HCV) infection from 2 hemodialysis centers accepted sofosbuvir based treatment as we reported previously. Serum potassium concentrations were tested every 4 weeks or on demand. Ten of 33 patients showed recurrence of hyperkalemia. We summarized the characteristics of hyperkalemia occurrence in these 10 patients. Overall, 24 episodes of hyperkalemia were observed in these 10 patients, 21 were under treatment and 3 were after treatment. Patients with or without hyperkalemia before sofosbuvir treatment didn’t show significantly differences in the median frequencies of hyperkalemia episodes during the observation period (3.5 vs. 2, *p* = 0.264).

**Conclusions:**

Patients with stage 5 CKD and HCV infection treated with sofosbuvir based regimens, even halved sofosbuvir, should be taken caution and closely monitoring serum potassium and renal function is necessary.

## Background

Hepatitis C virus (HCV) infection is a leading cause of chronic liver disease, cirrhosis, and hepatocellular carcinoma. Patients with chronic kidney disease (CKD), especially those with end-stage renal disease (ESRD) on hemodialysis, present high prevalence of HCV serum positivity. Currently, since the high efficacy and well tolerance to direct antiviral agents (DAAs), antiviral treatment is recommended for all patients infected with HCV, including those with stage 4–5 CKD on hemodialysis and waiting for renal transplantation [1].

Studies showed that sofosbuvir based regimens were highly efficacy and well tolerance in patients with HCV and stage 4–5 CKD [2, 3]. However, the safety of sofosbuvir, mainly sofosbuvir-derived metabolites in patients with CKD remains an opening question. In the safety pharmacology studies of sofosbuvir, high dose of GS-9851 (the parent of sofosbuvir) inhibited potassium current by approximately 13% at 159 μg/mL [4]. Patients with CKD are more prone to imbalance of homeostasis, such as metabolic acidosis, hyperkalemia. Based on our knowledge, up to now, serum potassium in patients with CKD treated with sofosbuvir has not been reported.

Previously, we reported a nosocomial HCV outbreak in Zhen’an County Hospital, Shaanxi Province, China, in January 2016 [5]. Thirty-three patients with hepatitis C and stage 5 CKD on hemodialysis were treated with half dose of sofosbuvir (200 mg) and full dose of daclatasvir (60 mg). All patients completed 24-week treatment and additional 12-week follow-up. This special population provided a unique opportunity for us to observe the serum potassium concentration during sofosbuvir treatment. Serum potassium concentrations were regular tested every 4 week or on demand during the treatment course and post-treatment up to 12 weeks. The levels of serum potassium 3 months prior to treatment were retrieved from the medical record database of the hospitals. Hyperkalemia was defined as serum potassium concentration higher than 5.5 mmol/L. Ten out of 33 were reported recurrence of hyperkalemia. We report hyperkalemia occurrences of these 10 patients with CKD and HCV infection who received sofosbuvir-based regimens.

## Case presentation

### Baseline characteristics

General information, treatment regimens, comorbidity, dialysis history, co-medication and adverse effects (AEs) were retrieved form electronic medical record. Means were used to describe quantitative variables and medians were used to describe the number of hyperkalemia episodes. All statistical analyses were performed using SPSS 16.0. Patients’ demographics were presented in Table 1. There were 7 males and the average age was 49.2 ± 14.5 years old. The average dialysis period was 2.85 ± 1.17 years. Drug-drug interaction was searched in HEP Drug Interactions database reserved by the University of Liverpool (data from the website: http://www.hep-druginteractions.org). The medicines combined with DAAs in these 10 patients do not present clinically significant interaction with sofosbuvir. Seven patients took DAAs in combination with at least one of the following medicines, nifedipine, carvedilol and amlodipine, which is reported potential interaction with daclatasvir. Carvedilol is contraindicated in patients with severe liver impairment. All of the patients were mild liver damage and the liver function returned normal during the antiviral treatment.

**Table 1:** Demographics of the 10 patients at baseline

Patient	Gender (F/M)	Age (Years)	Underlying Diseases	#Dialysis History	Co-medication
No. 1	M	72	Diabetes, Hypertension	3 years	atorvastatin, nifedipine, metoprolol tartrate, caltrate, insulin
No. 2	M	56	Diabetes, Hypertension, Nephrolithiasis, Coronary heart disease	4 years	nifedipine, carvedilol, novolin 30R
No. 3	F	56	Diabetes, Hypertension	4 years	nifedipine, metoprolol tartrate, carvedilol, novolin 30R
No. 4	M	57	Nephritis, Hypertension	4 years	nifedipine, metoprolol tartrate, carvedilol, amlodipine
No. 5	F	54	Nephritis, Hypertension	4 years	nifedipine, metoprolol tartrate, carvedilol
No. 6	M	57	Obstructive Nephropathy, Hydronephrosis, Chronic hepatitis B	1.5 years	None
No. 7	M	45	Hydronephrosis	2 years	metoprolol tartrate, vitamin B12, folic acid
No. 8	M	43	Nephritis; Hypertension	1 years	nifedipine, metoprolol tartrate, carvedilol
No. 9	F	27	Diabetes, Hypertension	3 years	nifedipine, amlodipine, metoprolol tartrate, caltrate, calcitriol, vitamin B12, folic acid, polysaccharide-iron complex
No. 10	M	25	Nephritis, Hypertension Coronary heart disease, Chronic Hepatitis B	2 years	nifedipine, metoprolol tartrate, caltrate

The antiviral therapy was half dose of sofosbuvir (200 mg) daily or post-dialysis and full dose of daclatasvir (60 mg)

# Dialysis scheme was 5 times per 2 weeks for these patients with stage 5 chronic kidney disease

### Occurrence of hyperkalemia

**Fig. 1:**
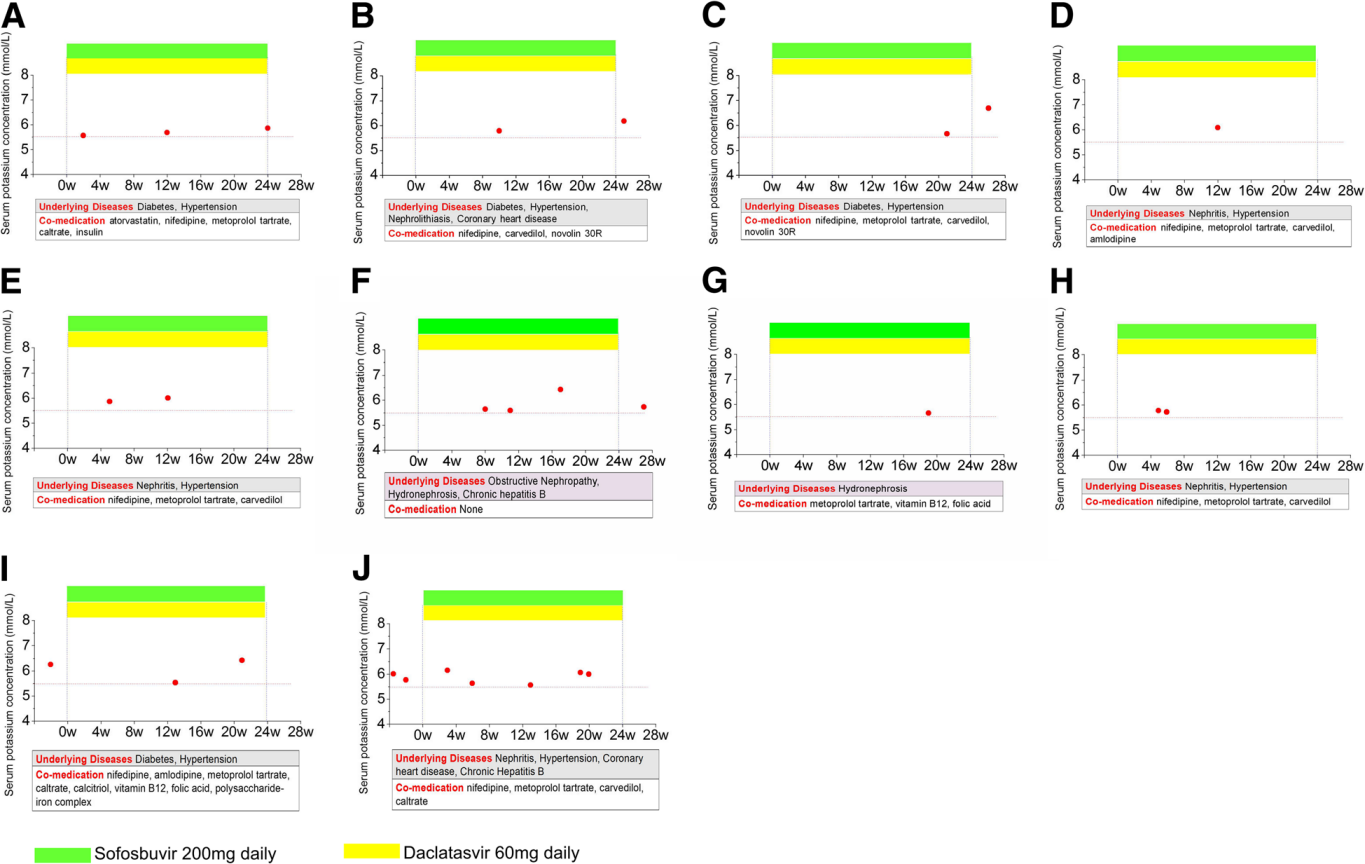
The detailed hyperkalemia of 10 patients. **a**-**j** present the hyperkalemia in patients No. 1-10, respectively

Figure 1 showed the detailed hyperkalemia occurrences of the 10 patients. Overall, 24 episodes of hyperkalemia were observed in these 10 patients. Of 24 episodes of hyperkalemia, 21 were observed during treatment and 3 were observed after the end of treatment. There were 2, 5, 5, 2, 4, 3 episodes of hyperkalemia in 0–4, 5–8, 9–12, 13–16, 17–20, 21–24 weeks, respectively. Three episodes of hyperkalemia were observed post-treatment at week 1, 2 and 3 post-treatment. In summary, hyperkalemia could occur at any time of the observational period from on-treatment to post-treatment.

Of the 10 patients, 2 showed history of hyperkalemia before antiviral treatment. Then, we tested if the history of hyperkalemia before treatment could increase the possibility of hyperkalemia on treatment. A total of 7 episodes of hyperkalemia were observed in the 2 patients with hyperkalemia history, while 17 episodes of hyperkalemia were observed in the rest of 8 patients without hyperkalemia history. Non-parametric test was used to compare hyperkalemia episodes between the patients with and without hyperkalemia history pre-treatment. The median frequencies of hyperkalemia episodes in patients with and without hyperkalemia history pre-treatment were 3.5 and 2, respectively. No significant differences between the patients with and without hyperkalemia history pre-treatment (*p* = 0.264).

## Discussion and conclusion

This is the first time to report recurrent hyperkalemia in patients with CKD and HCV infection treated with half-dose of sofosbuvir based regimen, although bradycardia, changed intracellular Ca^2+^ and acute interstitial nephritis have been reported potentially associated with sofosbuvir.

The recurrence of hyperkalemia was highly related to the administration of sofosbuvir. Patients with CKD especially in uremia period are at high risk of hyperkalemia, while regular dialysis aims to replace the function of kidney and could maintain the balance of electrolyte. In our case serials, we cannot attribute recurrence of hyperkalemia to insufficiently dialysis, for the dialysis scheme was unchanged before, during and after antiviral treatment. The dialysis therapy was sufficient for these 10 patients; because the serum creatinine levels remained stable and majority (8/10) of the patients were not observed hyperkalemia prior to sofosbuvir based treatment. Furthermore, other electrolytes, such as serum sodium or serum calcium disorder was not observed, and acid-base kept in homeostasis, supporting the adequacy of hemodialysis. Moreover, after the end of treatment, 8 out of the 10 patients did not present hyperkalemia, and along with the time extension of drug cessation, no hyperkalemia was observed. The temporal consistency between the DAAs usage and hyperkalemia occurrence highly supported the association between DAAs and hyperkalemia. Based on the HEP Drug Interactions database, no clinically significant interaction was showed between the co-medicines. Collectively, the raising levels of serum potassium were highly suspected in association to the usage of sofosbuvir.

The mechanism of the observed hyperkalemia in patient with CKD and HCV infection treated with sofosbuvir based regimens was unclear. As mentioned above, the recurrence of hyperkalemia could not be attributed to the insufficiency dialysis. Previously, case serials of bradycardia resulted from the co-administration of sofosbuvir and amiodarone were reported [6]. Regan CP et al. and Millard DC et al. demonstrated that co-administration of sofosbuvir and amiodarone produced dysfunction of the sinoatrial node automaticity and atrioventricular node conduction, decrease in cardiomyocyte mechanical activity and intracellular Ca^2+^ transients [7, 8]. Acute interstitial nephritis revealed by renal biopsy was reported potentially related to sofosbuvir, we assume that potassium exchange between inside and outside of cells may be an important factor of hyperkalemia [9, 10]. Actually, although no literature reports the impact on potassium channel by sofosbuvir, high dose of GS-9851 (the parent of sofosbuvir) did inhibit hERG-mediated potassium current in HEK293 cells expressing cloned hERG channels [4]. Integrating these evidences together, sofosbuvir do induce the dysfunction of ion channel, and such impact is more prominent in those cases with CKD and co-administered with drugs acting on an ion channel. In current report, calcium-channel blockers (CCBs) were administered in 7 patients. The drug-drug interaction could not be neglected in patients with stage 5 CKD co-administered with sofosbuvir and CCBs, since the metabolic pathway of CCBs have an intersection with that of sofosbuvir.

This is a retrospective report, the occurrence of hyperkalemia may be underestimated especially in patients with stage 5 CKD. However, the observation of this report could still provide a clue for clinicians to strengthen the monitoring for serum potassium when treating HCV infection with sofosbuvir, even its dose is halved, based regimens in patients with CKD.

## Data Availability

The datasets used during the current study are available from the corresponding author on reasonable request.

## Abbreviations

AEs: Adverse effects
CCBs: Calcium-channel blockers
CKD: Chronic kidney disease
DAAs: Direct antiviral agents
ESRD: End-stage renal disease
HCV: Hepatitis C virus
